# Dietary Supplementation with Organic Acids Improves Production Performance and Intestinal Health of Largemouth Bass

**DOI:** 10.3390/ani16081198

**Published:** 2026-04-15

**Authors:** Chaoran Ma, Yang Xiao, Shengquan Xiong, Jiao Yu, Wenyan Chen, Yuanfa He, Yongjun Chen, Shimei Lin

**Affiliations:** 1 College of Fisheries, Southwest University, Chongqing 400715, China; 17838407781@163.com (C.M.);; 2 Key Laboratory of Freshwater Fish Reproduction and Development (Ministry of Education), Southwest University, Chongqing 400715, China; 3 Yongchuan District Agriculture and Rural Affairs Commission, Chongqing 402100, China

**Keywords:** organic acids, growth performance, intestinal health, gut microbiota, *Micropterus salmoides*

## Abstract

Organic acid supplementation in aquafeed plays a crucial role in enhancing fish health and performance, contributing to sustainable aquaculture practices amid growing global demand for protein sources. In a feeding trial lasting 8 weeks, 360 juvenile fish (43.5 ± 0.23 g) were randomly assigned to four groups (three replicates per group; 30 fish per replicate), following one of four dietary regimens: the control (CON), or the control diet supplemented with 0.3% citric acid, 0.3% fumaric acid, or 0.3% malic acid. The supplementation of diets with the three organic acids significantly promoted weight gain (WG) and specific growth rate (SGR). Additionally, a significant increase in the activities of serum antioxidant enzymes (SOD, CAT, AKP) was recorded, with concomitant decreases observed in hepatic parameters (TG, GLU, ALT, AST) and the serum lipid peroxidation product MDA. Similarly, the organic acids supplementation also enhanced the hepatic antioxidant capacity (CAT, T-AOC, GSH-PX) and their gene expression levels, and decreased hepatic lipid and glycogen levels. Additionally, dietary organic acid supplementation significantly enhanced the activities of both digestive and antioxidant enzymes in the intestine. Furthermore, it improved intestinal morphology, as evidenced by increases in villus height, villus width, and muscular thickness. Moreover, supplementation with organic acids improved intestinal permeability, mediated through the suppression of serum DAO activity and LPS levels, accompanied by upregulated intestinal expression of junction complex components (Claudin-1, ZO-1, Occludin-1) and downregulated pro-inflammatory mediators (*tnf-α*, *il-1β*). The 16S rRNA sequencing demonstrated that CA induced a significant shift in the intestinal microbiota composition, marked by an elevated relative abundance of Firmicutes (including *Streptococcus*) and *Acinetobacter*, along with a decreased abundance of *Aeromonas*. These findings demonstrate that organic acids may enhance fish growth performance and intestinal health through their modulating effects on gut microbiota, intestinal development, immune responses, and antioxidant capacity. Notably, the dietary CA supplementation exhibited the most pronounced efficacy.

## Simple Summary

This study addresses the issues of slow growth and poor health in fish farming, showing that adding organic acids to feed can improve the performance of largemouth bass. Notably, one type of acid had the most significant effect. This finding provides practical insights for aquaculture production, and using this acid could be a method to boost fish yields. Through this approach, the farming industry can produce healthier fish, reduce losses, support people in obtaining reliable food sources, and promote environmentally friendly agriculture.

## 1. Introduction

Advances in intensive aquaculture practices have enabled remarkable increases in fish production growth rates [1,2], thereby helping to satisfy a critical need for animal protein in the human diet [3]. However, the intensification of aquaculture is associated with a considerable metabolic burden on fish [4], along with the induction of diverse stressors and inflammatory pathways [5]. These factors increase the susceptibility of fish to intestinal disorders, ultimately hindering growth and reducing overall productivity [5,6]. There is consensus that intestinal health is a cornerstone of efficient production, general well-being, and the ability to resist microbial infections in farmed fish [6]. Therefore, it is essential to improve intestinal health and enhance disease resistance through innovative and efficient approaches in the modern aquaculture industry [7]. Recently, the capacity of organic acids (or acidifiers) to support gastrointestinal health and enhance nutrient absorption has established them as a focus of significant research interest.

Owing to their antimicrobial properties, capacity to enhance gut health, and improvement of feed efficiency, organic acids (OAs) have versatile applications. These range from serving as growth promoters in animal husbandry to ensuring the hygiene of food-contact surfaces in the industry [8,9,10,11]. The commonly used OAs include citric acid (CA), fumaric acid (FA), and malic acid (MA). Recent studies have shown that appropriate supplementation of these OAs can generate beneficial impacts on nutrient utilization, gut health, and microbiota, enhancing feed utilization and growth in aquatic species [12,13] and a terrestrial animal [14,15], although outcomes may vary depending on acid type, dosage, and species-specific factors. This is because acids like CA and MA readily permeate cell membranes and exert antimicrobial effects largely through intracellular acidification and metal chelation [16,17], while CA and MA primarily inhibit bacteria by reducing pH and chelating metals, FA—which is derived from succinate oxidation in the TCA cycle and converted to malate—exerts its antimicrobial effect largely through its high pKa and lipophilic nature, facilitating penetration into bacterial cells and subsequent intracellular acidification [18,19,20]. Nevertheless, the effectiveness of OAs is inconsistent across studies, and the anticipated positive outcomes are not always observed. Several key determinants, including but not limited to feed formulation, acid characteristics (type and dosage), selected response variables, and fish species, collectively influence OA efficacy.

Largemouth bass (*Micropterus salmoides*) ranks among the most significant farmed fish species in China, valued for its rapid growth, palatable flesh, and high economic return, with its annual production surpassing 880,000 tons in 2023 [21]. Likewise, under adverse stimuli such as microbial infection, the cultivation of largemouth bass is frequently compromised by a range of intestinal pathologies, including oxidative stress, inflammation, disruptions to the microbiota [22], high-density feeding [23,24], and heat stress [25] of the external environment. This results in economic loss and a lack of sustainability. Therefore, it is essential to enhance rearing conditions and intestinal health to prevent diseases. Intestinal health is crucial for largemouth bass, and studies have shown that regulating the intestinal microbiota of largemouth bass can improve their intestinal health and growth performance [26,27]. However, systematic comparisons of different organic acids (OAs) in terms of their effects on multiple physiological parameters in carnivorous fish remain limited, and their mechanistic basis is not fully defined. To address this gap, the present study was designed to systematically compare the effects of dietary supplementation with three organic acids—citric acid (CA), fumaric acid (FA), and L-malic acid (MA)—at the same inclusion level of 0.3% on the growth performance of largemouth bass (*Micropterus salmoides*). We hypothesize that these three OAs will exert differential effects on growth performance in this carnivorous species, thereby revealing their relative efficacy through direct parallel evaluation. This study further aimed to assess their relative impacts on intestinal development, digestive and antioxidant enzyme activities, barrier function, and inflammatory responses, and to evaluate their influence on gut microbiota composition, ultimately offering practical insights for the application of organic acids in largemouth bass aquaculture and fishery production.

## 2. Materials and Methods

### 2.1. Diet Preparation

All experimental diets were carefully designed to maintain equal nitrogen levels at 49% crude protein and consistent fat content at 11% crude fat (Table 1). The control group received a diet without any organic acid additives. Three specialized treatment diets were created by adding either 0.3% citric acid (CA), fumaric acid (FA), or L-malic acid (MA) to the base formula. Every organic acid used met a purity standard of 99.5% or higher. After being finely ground to 320 μm and thoroughly mixed, the feed mixture was formed into 3.0 mm pellets. These pellets were then left to air-dry at room temperature for 24 h, after which they were sealed in an airtight container and refrigerated at 4 °C until they were ready for use.

### 2.2. Animal Handling and Feeding Management

Southwest University’s Animal Ethics Committee ratified this research protocol (Approval No. IACUC-20181015–12).

The experiment utilized 360 juvenile largemouth bass (43.5 ± 0.23 g) procured from Chongqing Three Gorges Ecological Fishery Co., Ltd. (Chongqing, China). The specimens were then randomly distributed across four distinct dietary regimens, with each treatment consisting of three replicate tanks containing 30 fish each. This eight-week trial was carried out in a controlled indoor recirculating aquaculture system. The fish were fed twice daily at 8:00 a.m. and 8:00 p.m. at a rate of 2% of their body weight. Throughout the experiment, the aquatic environment was carefully maintained with dechlorinated water and kept under specific parameters: nitrite levels remained below 0.01 mg/L, ammonia-N concentrations stayed under 0.1 mg/L, water temperature fluctuated between 22 and 28 °C and was identical across all experimental tanks (monitored daily with no significant inter-group differences, *p* > 0.05); this range falls within the optimal temperature for largemouth bass growth and metabolism, and therefore did not require statistical control as a covariate in the analyses, and dissolved oxygen levels never dropped below 6.0 mg/L. The lighting schedule followed a 12 h cycle, with illumination from 8:00 a.m. to 8:00 p.m. each day.

### 2.3. Sample Collection and Preparation

Once the eight-week experimental period concluded, all fish were subjected to a 24 h fasting period before being anesthetized for sample collection. Each specimen was carefully weighed, body length was precisely measured, and survival counts were documented. From each group, nine fish were randomly chosen to provide blood samples for subsequent biochemical analysis.

The visceral mass, liver, intestinal tract, mesenteric fat, and dorsal white muscle tissues were collected and weighed accurately. Liver and intestinal tissues were washed twice with PBS buffer and photographed for recording. Corresponding tissue samples from 6 fish in each group were fixed in 4% paraformaldehyde solution for paraffin embedding and histological section analysis. In addition, approximately 5 mm^3^ of liver tissue and 1 cm of intestinal segments (foregut and hindgut) were rapidly frozen in liquid nitrogen and then transferred to RNAlater^®^ (Thermo Fisher Scientific, Waltham, MA, USA) or DNA stabilization solution at 4 °C overnight. After 24 h, the samples were transferred to −80 °C for long-term storage. Intact gut tissues were used for microbial community analysis. Liver intestinal tissues were subjected to chemical analysis, histological examination, enzyme activity measurement, and gene expression analysis. In addition, dorsal muscles were subjected to chemical and texture analyses.

### 2.4. Measurement of Indicators

#### 2.4.1. Growth Performance and Body Indicators

At the end of the 8-week experiment, all surviving fish from each replicate tank were fasted for 24 h, anesthetized, and then weighed and measured. Initial body weight (IBW) and length were recorded at the start of the experiment, while final body weight (FBW) and length were recorded at the end. A digital balance (Jinnuo High Capacity Balance YP-200001D, Yuyao Jinnuo Balance Instrument Co. Ltd., Ningbo, China) and an ordinary measuring ruler were used for these measurements. The number of dead fish during the experiment was also recorded. These data were then used to calculate the following key growth and feed utilization parameters:

Weight Gain Rate (WGR, %) = [(Final body mass − Initial body mass)/Initial body mass] × 100

Specific Growth Rate (SGR, %/day) = [(Ln Final mass − Ln Initial mass)/Culture period (days)] × 100

Feed Efficiency Ratio (FCR) = Total Feed Intake/Wet Weight Gain

Daily Feed Intake (FI, g/fish/day) = Total Feed Consumed/[Average Fish Number × Days]

Survival Rate (SR, %) = (Final Fish Number/Initial Fish Number) × 100

#### 2.4.2. Determination of Conventional Nutrients

The conventional nutritional composition of whole fish and liver samples was analyzed according to internationally recognized standard methods. We determined moisture content by drying samples at 105 °C until they reached a stable weight. For crude protein analysis, we employed the Kjeldahl method to digest the samples and assess their nitrogen levels. Lipid extraction was performed using the Soxhlet technique with anhydrous ethyl ether as our solvent of choice. Finally, crude ash content was established by incinerating the samples in a muffle furnace at 550 °C until only white ash remained.

#### 2.4.3. Determination of Serum Biochemical Indexes

We obtained blood samples through tail vein puncture employing a disposable syringe rinsed with sodium heparin (1000 IU/mL) to prevent clotting. Excess heparin was expelled, leaving only the dead space of the syringe filled, which was then transferred to sterile, enzyme-free EP tubes (model EP-150X-J; Sangon Biotech Co., Ltd., Shanghai, China). These samples were allowed to sit at room temperature for a two-hour period to allow clotting and facilitate serum separation. Following this process, the serum was portioned into aliquots and preserved in an ultra-low temperature freezer set at −80 °C to prevent the detrimental effects of multiple freeze–thaw cycles. The collected serum was used for the determination of serum biochemical indexes (TP, GLU, TG, TC, AST, ALT, AKP). We utilized a HITACHI 7100 (ISE) automated biochemical analyzer (Hitachi High-Tech Corp., Tokyo, Japan) to carry out the determination process. The assessment of D-lactate content, endotoxin (ET) activity, and diamine oxidase (DAO) activity in all serum samples was conducted using the enzyme-linked immunosorbent assay (ELISA) technique. The operation and detection methods refer to the kit instructions (Nanjing Jiancheng Bioengineering Institute, Nanjing, China). To ensure measurement accuracy for largemouth bass serum, the ELISA kits (Shanghai Enzyme-linked Biotechnology Co., Ltd., Shanghai, China) were validated and calibrated according to the manufacturer’s protocols and standard aquaculture practices, including confirmation of parallelism (serial dilutions paralleled the standard curve), spike recovery (85–115%), and intra-/inter-assay coefficients of variation (<10%).

#### 2.4.4. Tissue Physiological and Biochemical Determination

Fresh liver and intestinal tissues were thoroughly blended with ice-cold saline at a 1:9 weight-to-volume ratio utilizing a mechanical tissue disruptor. Following this, the resulting mixture was subjected to centrifugation at 3500 times gravity for ten minutes while maintained at 4 degrees Celsius, at which point the clarified liquid layer was carefully extracted for further biochemical examination.

Commercial assay kits from Jiancheng BioEngineering Institute (Nanjing, China) were employed to quantify key antioxidant capacity parameters, including glutathione peroxidase (GSH-PX), total antioxidant capacity (T-AOC) via the FRAP method, catalase (CAT) using the ammonium molybdic acid technique, and total superoxide dismutase (T-SOD) through both the FRAP and WST-1 methods. Additionally, malondialdehyde (MDA) levels were determined using the thiobarbituric acid (TBA) method. Meanwhile, intestinal enzyme activities—including trypsin (UV colorimetry), lipase (colorimetric analysis), and amylase (starch iodine colorimetric method)—were all quantified with a microplate reader (Model Infinite M200 Pro, Tecan, Group Ltd., Männedorf, Switzerland).

#### 2.4.5. Histological Sections of Intestine and Liver

The liver and intestinal samples were fixed in 4% paraformaldehyde for 24 h at room temperature to preserve tissue structure. Following fixation, the tissues were dehydrated through a graded series of ethanol solutions (70% for 1 h, 80% for 1 h, 90% for 1 h, 95% for 1 h, and 100% twice for 1 h each) to remove water. The dehydrated samples were then cleared in xylene (two changes, 1 h each) to make the tissues transparent. Subsequently, the tissues were infiltrated with molten paraffin wax and embedded using a Kuohai Technology KH-BL embedding machine (KH-BL; Wuhan Kuohai Medical Technology Co., Ltd., Wuhan, China), which includes a cooling station to solidify the paraffin blocks rapidly. The paraffin-embedded tissues were sectioned at 4 μm thickness using a rotary microtome. The sections were mounted on glass slides, deparaffinized, rehydrated, and stained with hematoxylin–eosin (H&E) staining solution according to standard protocols (hematoxylin for 5 min, followed by eosin for 2 min). After staining, the sections were dehydrated, cleared, and coverslipped. The stained sections were observed and imaged under a light microscope (Nikon Eclipse E100, Nikon Corporation, Tokyo, Japan) at various magnifications to assess histological changes.

#### 2.4.6. Enteric Microorganism

The gut microbial samples (16S rRNA high-throughput sequencing) were sequenced, and three biological replicates were obtained per group. Microbial genomic DNA was isolated from intestinal content samples employing the PowerSoil DNA Isolation Kit (Mo Bio Laboratories, Carlsbad, CA, USA). The resulting sequencing data were processed on the BMKCloud platform (Beijing Biomarker Technologies Co., Ltd., Beijing, China.), with PCR amplification and sequencing performed on the Illumina HiSeq 2500 platform (Illumina, Inc., San Diego, CA, USA). OTU clustering was performed using USEARCH (version 10.0) at a 97% sequence similarity threshold (default), with OTUs filtered at a default threshold of 0.005% of the total sequenced reads. Profiling of the sample communities’ taxonomy was carried out at the phylum, class, order, family, genus, and species levels using QIIME2 (version 2022.2; https://qiime2.org) (classify–consensus–blast method, default top *N* = 3 alignments, minimum sequence similarity 90%, minimum coverage 90%, and minimum consensus 51%). The principal component analysis (PCA) diagram was drawn using R language tools (version 4.1.0; R Core Team, Vienna, Austria). Microbial alpha diversity was assessed in QIIME2 using indices including Chao1, ACE, Shannon, and Simpson, in addition to determining the OTU coverage rate. Metastats (version 20090414; University of Maryland, College Park, MD, USA) was then utilized to evaluate substantial differences in bacterial populations among groups: a T-test was performed on inter-group species abundance data to obtain *p*-values, followed by FDR correction to obtain q-values; differentially abundant taxa were screened based on *p*-values (or q-values) with the default threshold of *p* < 0.05.

#### 2.4.7. Relative Gene Expression

Total RNA was extracted using RNAiso Plus reagent (TaKaRa Bio Inc., Kusatsu, Shiga, Japan), and the concentration of the isolated RNA was subsequently determined by NanoDrop2000 ultramicro spectrophotometer (Thermo Fisher Scientific, Wilmington, DE, USA), and then diluted to 1000 ng/μL. First-strand cDNA was synthesized from total RNA using the PrimeScript™ RT reagent Kit with gDNA Eraser, diluted with DEPC water, and stored. The total reaction volume for the real-time quantitative PCR (qPCR) was 20 μL, which consisted of 2 μL cDNA, 1 μL of each upstream and downstream primers, 10 μL of BlasTaqTM2xqPCRMM, and enzyme-free water. The relative mRNA expression of the target gene was determined by normalizing its expression level to that of the internal reference gene, and the Ct value of each concentration of cDNA of each primer pair was obtained by quantitative PCR. The sequence of qPCR primers is shown in Table 2. The relative gene expression was computed mathematically via the 2^^−ΔΔCt^ method.

### 2.5. Statistical Analysis

All results are expressed as mean values ± standard error of the mean (SEM). Statistical analyses were performed using SPSS version 25.0 (IBM Corp., Armonk, NY, USA). Prior to analysis, the assumptions of one-way ANOVA were verified: normality of data distribution was assessed using the Shapiro–Wilk test, and homoscedasticity was checked with Levene’s test. No data transformations were applied, as the assumptions were met for all parameters. Differences among groups were evaluated using one-way ANOVA followed by Tukey’s post hoc test for multiple comparisons, with statistical significance set at *p* < 0.05. For gut microbiota analysis, relative abundances at the phylum and genus levels were compared between groups using Metastats in the R package (version 4.0.3). Unadjusted *p*-values are reported, as no multiple testing correction (e.g., FDR) was applied due to the exploratory nature of the microbiota comparisons and the limited number of tested taxa. Data visualization, including bar graphs and heatmaps, was performed using GraphPad Prism 8.0 (GraphPad Software, San Diego, CA, USA).

## 3. Results

### 3.1. Growth Performance and Body Composition

After the 8-week trial, fish fed the CA, FA, and MA diets demonstrated significantly enhanced growth, as indicated by higher FBW, WG, and SGR compared to the control (*p* < 0.05; Table 3), while no significant differences were observed in FCR and SR. All dietary groups exhibited similar whole-body compositions, with moisture, crude protein, and crude fat levels remaining statistically similar (*p* > 0.05). Compared with the control group, the crude ash content significantly increased in the experimental groups (CA, FA, and MA), while the liver lipid and glycogen contents significantly decreased (*p* < 0.05, Table 4).

### 3.2. Serum Biochemical Indices

Serum total protein, alkaline phosphatase, superoxide dismutase, and catalase activities were significantly elevated in fish fed organic acid-supplemented diets, whereas glucose, triglycerides, AST, ALT, and MDA levels were significantly reduced (*p* < 0.05). However, Statistical analysis revealed no significant variation in serum TC levels among the groups (*p* > 0.05, Table 5).

### 3.3. Liver Histomorphology and Antioxidant Capacity

**Figure 1:**
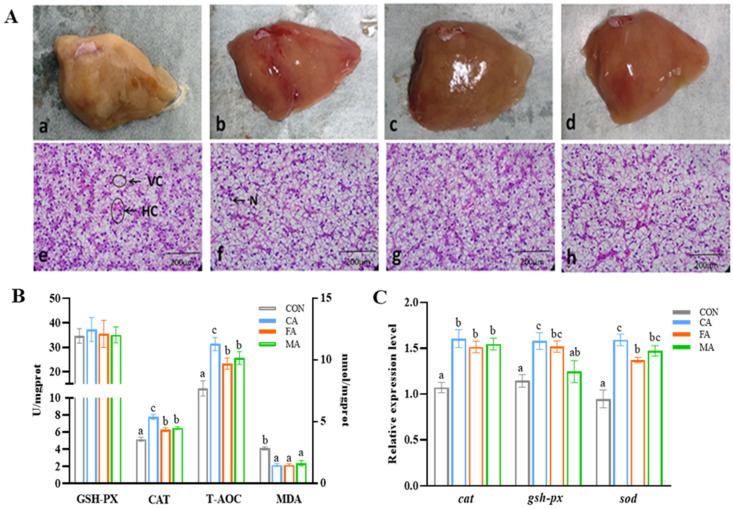
Effects of three organic acids on liver histology and antioxidant capacity of largemouth bass: (**A**) liver morphology and histology; (**B**) antioxidant enzyme activities; (**C**) antioxidant gene expression levels. (**a**) Control group, (**b**) MG group, (**c**) MB group, (**d**) ME group (liver appearance); (**e**) Control group, (**f**) MG group, (**g**) MB group, (**h**) ME group (H.E 200×). N: nucleus; VC: vacuole; HC: hepatic cords; GSH-PX: glutathione peroxidase; CAT: catalase; T-AOC: total antioxidant capacity; MDA: malondialdehyde. Data are expressed as mean (SEM, n = 3). Bars without letter or sharing the same letter represent no significant difference by Tukey’s test (*p* > 0.05).

Livers from the control group appeared pale, whereas those from fish fed organic acid-supplemented diets exhibited a healthier reddish color (Figure 1A). HE staining revealed that hepatocytes in the organic acids group exhibited normal morphology, characterized by orderly arrayed cells, significantly reduced vacuoles, and a clearly located cell nucleus in the center.

Results from the present study confirmed that dietary organic acid supplementation significantly enhanced hepatic antioxidant activities (CAT and T-AOC), along with a reduction in the lipid peroxidation product MDA, and the highest antioxidant capacity was observed in the CA group (*p* < 0.05, Figure 1B). The analysis revealed no significant differences in the activity of glutathione peroxidase among the groups (*p* > 0.05). Furthermore, the relative expression levels of *cat*, *sod*, and *gsh-px* were significantly up-regulated in three organic acids (CA, FA, and MA) groups compared to the control group (*p* < 0.05; Figure 1C).

### 3.4. Intestinal Development

**Figure 2:**
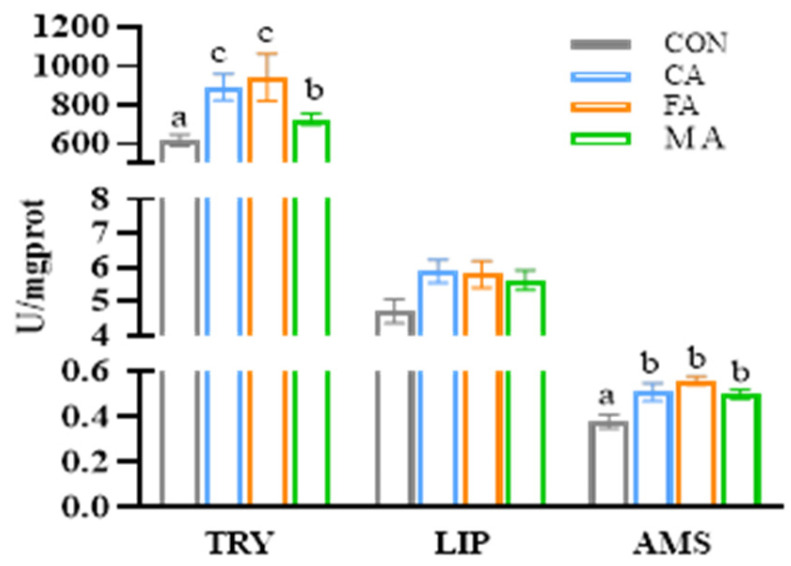
Effects of three organic acids on intestinal digestive enzyme activities of largemouth bass. TRY: trypsin; LIP: lipase; AMS: amylase. Data are expressed as mean (SEM, n = 6). Bars without letter or sharing the same letter represent no significant difference by Tukey’s test (*p* > 0.05).

**Figure 3:**
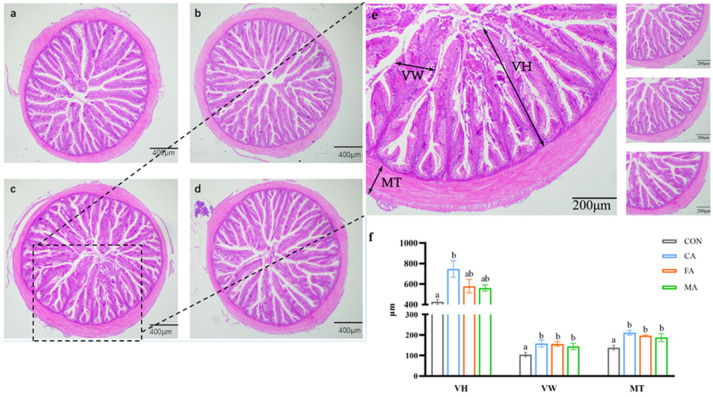
Effects of three organic acids on the midgut morphology in largemouth bass stained with HE (40 and 100×). (**a**) CON group; (**b**) CA group; (**c**) FA group; (**d**) MA group; (**e**) CA group, enlarged view. The three pictures on the right are the enlarged versions of (**a**,**b**,**d**). (**f**) Morphological quantitative indices. VH: villus height; VW: villus width; MT: muscular thickness. Data are expressed as mean (SEM, n = 3). Bars without letter or sharing the same letter represent no significant difference by Tukey’s test (*p* > 0.05).

Adding CA, MA, or FA significantly enhanced trypsin (TRY) and amylase (AMS) activities in the intestine (*p* < 0.05). However, supplementation with three organic acids did not remarkably affect the activity of lipase (LIP) in the intestine (*p* > 0.05, Figure 2). Further revealed that the midgut structure remained complete with neat villi and smooth mucosa among all groups. Relative to the control, the organic-acid-supplemented groups exhibited a significant increment in villus width (VW) and muscularis thickness (MT). Specifically, villus height (VH) was greatest in the CA-supplemented group compared to all other treatments (*p* < 0.05; Figure 3).

### 3.5. Intestinal Barrier Function

**Figure 4:**
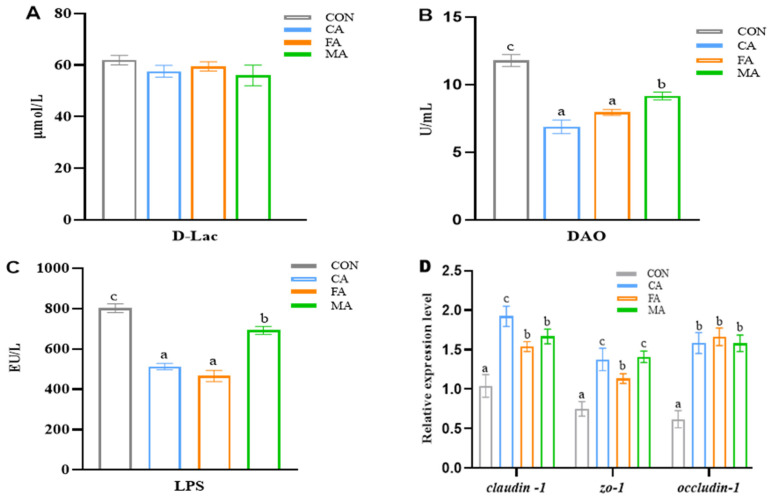
The parameters of intestinal permeability (**A**–**C**) and the expression levels of tight junction-related genes (**D**) in the intestine of largemouth bass fed the experimental diet. Data are expressed as mean (SEM, n = 6). Without letter or bars shared the same letter represents no significant difference by Tukey’s test (*p* > 0.05).

Compared to the control group, the CA, MA, and FA groups significantly reduced diamine oxidase (DAO) and lipopolysaccharide (LPS) levels in serum (*p* < 0.05, Figure 4B,C), while no significant difference was observed in D-lactic acid (D-Lac) among all groups (*p* > 0.05, Figure 4A). It was further found that three organic acids (CA, FA, and MA) supplementation significantly up-regulated *Claudin-1*, *ZO-1*, and *Occludin-1* mRNA expression levels (*p* < 0.05, Figure 4D) in the intestine.

**Figure 5:**
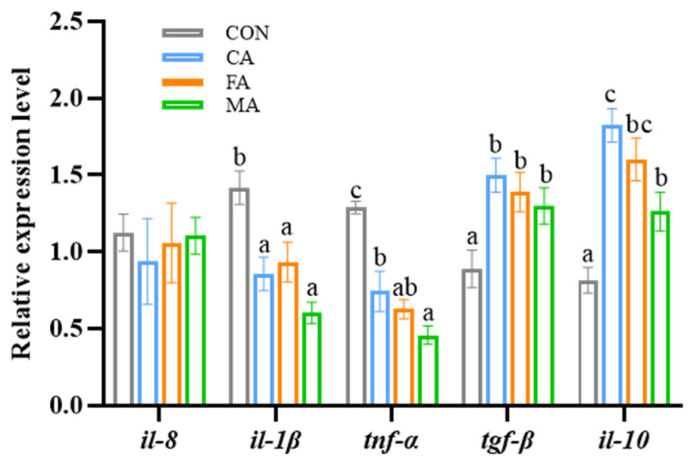
The expression levels of inflammatory cytokine genes in the intestine of the largemouth bass. il-8: interleukin-8; il-1β: interleukin-1β; tnf-α: tumor necrosis factor–α; tfg-β: transforming growth factor-β; il-10: interleukin-10. Data are expressed as mean (SEM, n = 9). Bars without letter or sharing the same letter represent no significant difference by Tukey’s test (*p* > 0.05).

**Figure 6:**
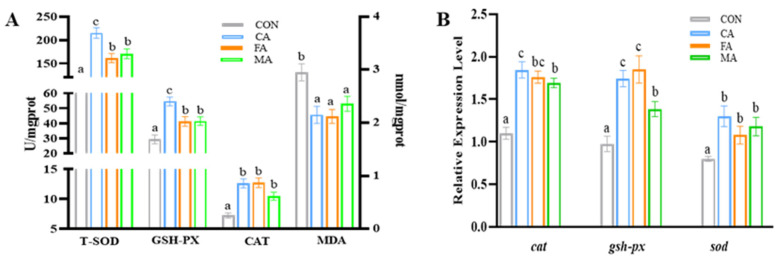
The antioxidant abilities (**A**) and antioxidant-related gene expression levels (**B**) in the intestine of largemouth bass. Data are expressed as mean (SEM, n = 6). Bars without letter or sharing the same letter represent no significant difference by Tukey’s test (*p* > 0.05).

The inflammatory status in the intestine was modulated by organic acid supplementation, as evidenced by a significant downregulation of pro-inflammatory cytokines (*il-1β* and tnf-α) and a concurrent upregulation of anti-inflammatory mediators (*tgf-β1* and *il-10*). Conversely, the expression of *il-8* remained unaltered (Figure 5). In parallel, the intestinal oxidant status was significantly improved, with activities of the antioxidant enzymes T-SOD, GSH-Px, and CAT being elevated, while the lipid peroxidation product MDA was reduced (Figure 6A). This enhancement at the enzyme level was supported by a corresponding upregulation in the mRNA expression of their respective genes (*cat*, *gsh-px,* and *sod*) (Figure 6B). For all mentioned results, *p* < 0.05 unless otherwise stated.

### 3.6. Microbiota Analysis

**Figure 7:**
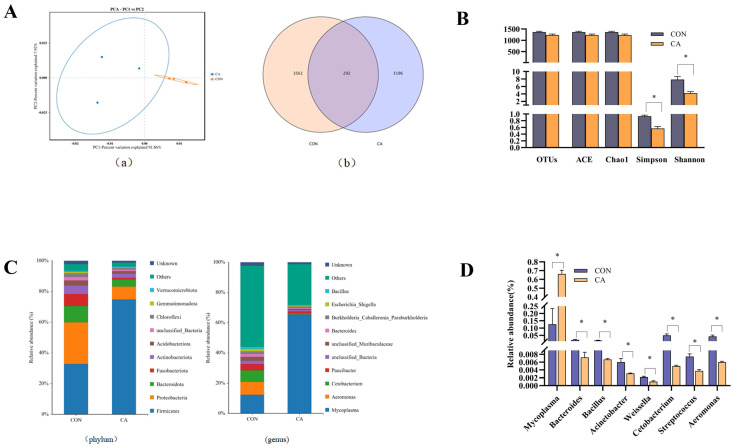
Effects of three organic acids on the intestinal microbiota of largemouth bass. (**A**) Principal component analysis (PCA) diagram of the intestinal microbiota (**a**) and OTU Venn diagram (**b**) (Supplementary Data S1B). (**B**) Effects of citric acid on the OTUs and α diversity of the intestinal microbiota of largemouth bass (Supplementary Data S1C). “*” indicate statistically significant differences (*p* < 0.05). (**C**) Effects of citric acid on the relative abundance of the intestinal microbiota of largemouth bass at the phylum level and genus level (Supplementary Data S1E). Data are presented as means ± SEM. (**D**) Metastats analysis of microbial community abundance differences (Supplementary Data S1D).

PCA revealed marked dissimilarities among the microbial communities of the control and CA groups (Figure 7(Aa)). Venn diagrams showed that the control and CA groups shared 292 identical core OTUs. Additionally, the CA group exhibited 3196 unique OTUs, whereas the control group contained 3561 unique OTUs (Figure 7(Ab)). Figure 7B showed that the CA group significantly reduced the Shannon and Simpson indices (*p* < 0.05). The completed raw data summary is provided as Table S1 in the Supplementary Materials.

At the phylum level, Firmicutes, Proteobacteria, Bacteroidota, Fusobacteriota, and Actinobacteria were the dominant phyla, collectively accounting for more than 85% of the entire phylum. Notably, the relative abundance of Firmicutes increased from 32.72% to 74.79%, whereas Proteobacteria decreased from 27.14% to 8.37% in the CA group (Figure 7C). At the genus level, the top three microorganisms in both groups were *Mycoplasma, Aeromonas*, and *Cetobacterium*. Remarkably, the abundances of *Aeromonas* (from 8.50% to 0.19%) and *Cetobacterium* (from 7.56% to 0.79%) decreased, while *Mycoplasma* increased (from 12.37% to 65.42%). Metastats analysis confirmed that the relative abundance of *Aeromonas* and *Streptococcus* in the CA group was significantly lower than that of the control group (*p* < 0.05). Likewise, CA supplementation in the diet also decreased the abundances of *Cetobacterium*, *Bacillus*, and *Bacteroides* compared to the control group (*p* < 0.05, Figure 7D).

## 4. Discussion

Growth performance constitutes the principal determinant of economic returns in aquaculture. In this study, dietary organic acid supplementation enhanced the growth performance of largemouth bass without affecting the feed conversion ratio, likely due to improved feed utilization. Consistent with the present results, several studies have documented growth-promoting effects of dietary organic acids in other aquatic species, including *Sciaenops ocellatus* [28] and *Pelteobagrus fulvidraco* [29]. Organic acids also enhanced performance among terrestrial livestock, with particular emphasis on swine and poultry [3,30]. Furthermore, the findings of the current study also discovered that the OAs supplementation improved intestinal digestive enzyme activities, as reported in *E. sinensis* [31] and *S. ocellatus* [13]. These findings suggest that the positive impact of OA supplementation on growth performance may be partly due to the enhancement in digestive enzyme activity. However, contrasting results exist in the literature, with some studies reporting neutral to adverse outcomes on growth performance from OA supplementation [32]. Likewise, dietary MA and LA cannot promote the growth of *E. sinensis* [25]. These results led to the proposal of a hypothesis that the efficacy of OAs may vary depending on organic acid type and dose, intestinal development, the feed ingredients, the feeding environment, and animal strains [8,33].

Organic acid supplementation led to elevated whole-body ash content in the current study. This may imply that organic acids may improve mineral absorption and retention from the diet. However, without direct mineral balance markers, this remains a hypothesis that warrants further validation through targeted assays in future studies. These results were consistent with the findings of *E.sinensis* [31], which indicated that dietary OAs in increasing the ash contents were mainly ascribed to promoting the Ca and P accumulation. Similarly, MA supplementation added Ca and P contents in the vertebrae of *Cyprinus carpio* [34]. Earlier research documented an enhancement of Zn content in rainbow trout following the addition of CA to their feed [35], and P and Zn contents in the whole body of the large yellow croaker, *Larimichthys crocea* [36]. In addition, CA dietary acidification enhances mineral utilization and deposition in broiler chickens [37]. These findings showed that dietary OAs could regulate the availability of minerals, which might be the reason OAs promoted the growth of *M. salmoides*. In addition, dietary OAs supplementation can influence lipid metabolism [38,39]. Several studies have demonstrated that dietary malic acid (MA) supplementation can decrease whole-body lipid deposition in *Oreochromis niloticus* [40], and CA and FA also decreased the lipid content of the whole body [41]. Likewise, the present study showed that the OA groups had significantly lower liver lipid content than the control group. Thus, we inferred that dietary OAs reduced the lipid content in the liver, possibly through promoting mineral utilization and improving lipid metabolism, which deserves further investigation.

The current study also observed that three organic acids elevated antioxidant enzyme activities and attenuated MDA levels in liver and serum, suggesting that dietary OAs can reinforce the antioxidant capacity of *M. salmoides.* These results were in agreement with the findings of *Scophthalmus maximus* [42] and *O. niloticus* [43]. Moreover, the gene expression of *cat, sod,* and *gsh-px* was upregulated in the OAs-supplemented groups. This implies that OAs contribute to the mitigation of oxidative stress in *M. salmoides* via elevated expression of antioxidant-related genes. Interestingly, the addition of OAs reduced serum AST and ALT levels in this study, suggesting a protection of liver health. Our findings are consistent with a previous report in the MA-fed *O. niloticus* [40]. In contrast, the increase in organic acid concentration (combination of CA and MA) increased serum ALT and AST activities of *C. auratus gibelio* [44]. Likewise, adding high doses of MA (0.5 and 1% MA) in *O. mykiss* feeds and overfeeding for >8 weeks may cause liver injury [45]. The observed discrepancies in experimental outcomes can be largely accounted for by variations in OA dosage and the specific fish species studied. And more importantly, there were significant decreases in the content of TG and GLU in serum when dietary OAs were supplemented. Elevated plasma triglyceride (TG) levels, which are recognized to promote fat deposition [46], suggest that OAs may improve growth in the current study by modulating glucolipid metabolism, reducing hepatic lipid accumulation, and thus enhancing the efficiency of energy utilization for production.

To further substantiate the growth performance outcomes, we examined intestinal morphology. The OA groups exhibited concomitant improvements in key midgut metrics, specifically VH, VW, and MT. This positive influence on gut architecture is consistent with established knowledge, echoing observations in chickens where organic acids enhance intestinal structure [15,47]. Typically, LPS, D-Lac, and DAO are effective biomarkers to reflect intestinal mucosal injury. The present study found that serum DAO and LPS levels decreased with OA supplementation, but D-lactate did not differ. However, the reductions in DAO and LPS suggest improved intestinal barrier integrity and reduced bacterial endotoxin translocation, whereas the lack of change in D-lactate indicates that the protective effects of organic acids may primarily target mucosal integrity and Gram-negative bacterial leakage rather than all aspects of bacterial metabolite permeability; this nuanced pattern highlights the multifaceted regulation of gut barrier function and warrants further investigation into microbiota–metabolite interactions. Moreover, tight junctions are located at the epithelial cell apex, mainly regulating paracellular permeability and maintaining gut barrier integrity [48]. These cellular connections comprise architectural elements like occludin and claudin alongside functional components such as ZO-1 [49]. Subsequent investigations have further revealed that OA supplementation boosts intestinal production of tight-junction proteins, specifically Claudin-1, ZO-1, and Occludin-1. Moreover, organic acids have demonstrated their ability to elevate tight junction (TJ) protein levels across various species [15,50]. Our findings suggest that the growth-promoting advantages of OAs stem from improvements in intestinal structure and barrier integrity, with tight junction protein enhancement serving as the key evidence. The underlying mechanisms likely involve superior nutrient assimilation, presumably owing to the promoted development of midgut villi.

A key function of the mucosal immune system is to maintain immunological homeostasis and safeguard the functional integrity of the mucosal barrier [51]. The present study demonstrated that supplementation with three OAs improved the antioxidant capacity of largemouth bass in the intestine, as evidenced by increased T-SOD, GSH-Px, and CAT activities and decreased MDA content, and exhibited expression of *cat, gsh-px,* and *sod* levels, suggesting that OAs improved the oxidative status of the intestine. A similar study reported that dietary CA significantly enhances antioxidant capacity [52]. These findings demonstrate that the antioxidant benefits conferred by OAs are dose-dependent, with efficacy confined to a specific optimal range. Specifically, cytokines are recognized regulators of intestinal tight junctions [53]. Among these regulators, IL-1β and TNF-α are well-established for their role in increasing intestinal epithelial permeability [54], while TGF-β1 has opposite effects by raising transepithelial electrical resistance [55]. In this study, dietary OAs supplementation significantly decreased mRNA expression levels of pro-inflammatory cytokine *il-1β* and *tnf-α*, while markedly elevating the transcript abundance of anti-inflammatory cytokine *tgf-β1* and *il-10*. This exhibited that OAs had exerted anti-inflammatory activity and reinforced intestinal immune competence in largemouth bass. Therefore, these findings suggest that OAs supplementation may contribute to the alleviation of intestinal inflammation through the maintenance of TJ homeostasis. These data further demonstrated that OAs effectively enhanced the growth in largemouth bass by modulating intestinal health.

A dynamic reciprocity between the intestinal microbiota and the mucosal barrier is crucial for sustaining homeostasis within the gut ecosystem [56]. Studies have shown that OAs may improve animal intestinal homeostasis by regulating the gut microbiota structure, subsequently enhancing intestinal health and performance [57]. We speculate that organic acids exert multifaceted effects on gastrointestinal physiology through their concerted action on pH regulation, bacterial membrane depolarization, and intracellular acidification, ultimately leading to selective modulation of the gut microbiota. In this experiment, a diet supplementation with CA can remodel the microbiota, predominantly through an increased Firmicutes proportion with a concurrent reduction in both Bacteroidetes and Proteobacteria in largemouth bass. The Firmicutes phylum takes the lead in producing short-chain fatty acids, with an added benefit of helping to maintain the integrity of our gut lining [58]. On the other hand, Proteobacteria can serve as a red flag for dysbiosis and increased susceptibility to illness [59]. These results support the concept that CA-induced amelioration of gut flora contributes to its anti-inflammatory potential within the intestinal environment. Moreover, the ratio of Firmicutes/Bacteroidetes (FBR) increased from 3.06 to 15.71 in this study. An elevated Firmicutes-to-*Bacteroides* (F/B) ratio is indicative of enhanced nutrient absorption efficiency and metabolic capacity [60], also as a marker of metabolic alterations in humans and mice [61]. These findings indicate that the enhanced growth of largemouth bass following CA supplementation likely resulted from improved nutrient digestion and absorption. Notably, while Firmicutes and Bacteroidetes typically dominate gut microbiota composition, Proteobacteria were found to be the predominant phylum in largemouth bass. Thus, we introduced the novel Firmicutes/Proteobacteria (FPR). The ratio of FPR increased from 1.21 to 8.94 in this study, which exhibited a consistent increase from the CA group vs the control group. This correlation may serve as a reliable indicator for assessing the risk of intestinal microbial dysbiosis in clinical settings. As demonstrated in this study, with the improvement of growth, the authors observed an increase in FBR and FPR. Notably, the substantial elevation in FPR relative to FBR indicates that it represents a more accurate tool for evaluating the risk of microbial dysbiosis, a relationship that should be prioritized in future research.

Notably, significant changes in the gut microbiota in the CA group were also found at the level of genus. Our data show that adding CA results in a significant decrease in *Aeromonas*, *Streptococcus*, and *Acinetobacter*, which are regarded as potential opportunistic pathogens, positively correlated with gut permeability, and lead to intestinal dysfunction, causing inflammation [62]. Thus, for this study, CA was added to the diet of largemouth bass to further explore its impacts on host resistance to the potential pathogenic bacteria. These results indirectly suggested that CA inhibited the colonization of the potential pathogenic bacteria in the gut and maintained intestinal health. The body of research indicates that supplementing fish diets with organic acids substantially enhances the immune response of cultured fish against common pathogens such as *Streptococcus* agalactiae and *Aeromonas* hydrophila [63,64]. Findings from avian nutrition studies have shown that organic acids in feed effectively promote a healthy gut microbial ecosystem by selectively targeting harmful microbes while simultaneously fostering the growth of beneficial bacteria [57]. Collectively, the evidence points to a broader mechanism of action for organic acids, extending beyond direct antimicrobial effects to include mediation of gut microbial equilibrium. This rebalancing contributes to intestinal health, nutrient utilization, and overall performance. However, the specific causal links between OA-induced microbial shifts and opportunistic pathogen dynamics remain to be fully elucidated. and opportunistic pathogen. Surprisingly, CA supplementation in the diet also decreased the abundances of potential beneficial bacteria (*Cetobacterium*, *Bacillus*, and *Bacteroides*); this question could be addressed in a future study.

Collectively, our systematic assessment of three organic acids (citric, fumaric, and L-malic acids) in the diet of largemouth bass confirms their efficacy in enhancing growth performance through progressive improvements in feed digestibility, intestinal morphology, and antioxidant capacity. More precisely, organic acid supplementation reinforced the intestinal barrier via up-regulation of tight-junction proteins, improved digestive enzyme activity and intestinal immune function, and modulated gut microbiota composition. When growth performance and intestinal microbiota function were considered together, the citric acid (CA)-supplemented diet at 0.3% showed promising overall efficacy compared to the other organic acids tested, highlighting its potential to improve productivity in largemouth bass aquaculture. Moreover, the FBR and FPR ratios identified in this study represent potential novel biomarkers for predicting intestinal health risks, showing promise as diagnostic tools that warrant validation in future research. Nevertheless, since only a single inclusion level (0.3%) was tested, no definitive optimal dose or dose–response relationship can be inferred; therefore, further dose–response experiments are needed to validate and optimize this supplementation strategy for sustainable largemouth bass farming.

## 5. Conclusions

The dietary supplementation of three organic acids (citric acid, fumaric acid, and L-malic acid) significantly enhanced the antioxidant capacity of the liver and intestine, improved the intestinal physical barrier, and modulated the intestinal microbiota, thereby promoting the growth of largemouth bass. These effects were dependent on the type of organic acid, with citric acid demonstrating the most pronounced positive impact on overall health and growth performance.

## Figures and Tables

**Table 1:** Feed formula and nutrient composition (% air-dry basis).

Items	CON	CA	FA	MA
**Ingredient**				
Steam fish meal	35	35	35	35
Chicken powder	12	12	12	12
Cottonseed protein concentrate	14	14	14	14
Wheat gluten	3	3	3	3
Soybean meal	15	15	15	15
Cassava starch	10	10	10	10
Fish oil	2	2	2	2
Soybean oil	4	4	4	4
Choline chloride	0.5	0.5	0.5	0.5
Vitamin premix	1	1	1	1
Mineral premix	1.5	1.5	1.5	1.5
Ca(H_2_PO_4_)_2_	1.5	1.5	1.5	1.5
Microcrystalline cellulose	0.5	0.2	0.2	0.2
Citric acid		0.3		
Fumaric acid			0.3	
L-Malic acid				0.3
**Proximate composition (%)**				
Crude protein	49.16	49.41	49.39	49.27
Crude lipid	11.31	11.27	11.21	11.24
Dry matter	91.45	91.52	91.48	91.50
Ash	11.82	11.86	11.85	11.84
Gross energy (MJ/kg)	19.62	19.68	19.67	19.66

Notes: vitamin premix (mg/kg), VA 18, VD3 5, VE 150, VC (350 g kg^−1^) 500, VB_1_ 16, VB_6_ 20, VB_12_ 6, VK_3_ 18, riboflflavin 40, inositol 320, calcium-D-pantothenate 60, niacinamide 80, folic acid 5, biotin 2, ethoxyquin 100, mineral premix (mg/kg): Na 30, K 50, Mg 100, Cu 4, Fe 25, Zn 35, Mn 12, I 1.6, Se 0.2, Co 0.8.

**Table 2:** Primers pair sequences for real-time PCR.

Gene	Forward Primer (5′-3′)	Reverse Primer (5′-3′)	(bp)	GenBank
*cat*	GGTGTTCACGGATGAGATG	GGAGAAGCGGACAGCAAT	178	119893048
*sod1*	TTTTGAGCAGGAGGGCGATT	CTGAGCACCTTGTCCGTGAT	258	119895678
*cusr*	ACACGACAGGTATGAGGTTGGT	TCTGGCTCTGGCTACAGTCACT	214	119899420
*gpx1a*	CGTTACACTGCCAAGGGACTCGT	GCCATTCCCTGGACGGACATAC	120	119886459
*claudin*	CCAGTTTCTCCTGCCGTTG	CAACCCAGCCAGGAAACAG	169	119898961
*ocel1*	CCTGCTCAGACTTCTTGCCG	CTGTTGGACCACTCACTGTCTTTC	99	119902247
*zo-1*	GGCAAGAACCACCAAGAGG	GCTGCGAAGACCACGAA	141	119893804
*tnf-α*	AAATAGTGATTCCTCAAGACGG	TGAACAGTATGGCTCAGATGG	126	119906688
*il-8*	TCCTGGCTGCTCTGGCTCTC	GGATGGCCCTCCTGTTAATGG	111	119892024
*tgf-β*	GGCAATGTAAGCGGTATGTC	CTTGGTGCTGTTGTAGAGGG	186	119882881
*il-1β*	CGTGCCAACAGTGTGAAGAC	TGGACAGAACAACGGGACTAC	193	119914255
*il-10*	GCCAGCAGCATCATTACCAC	AACCAGGACGGACAGGAGG	115	119885912
*eef1a1*	GTTGCTGCTGGTGTTGGTGAG	GAAACGCTTCTGGCTGTAAGG	156	119907150

**Table 3:** Effects of three organic acids on the growth performance of largemouth bass ^1^.

Items	CON	CA	FA	MA
Initial body weight (IBW, g)	43.50 ± 0.13	43.35 ± 0.08	43.15 ± 0.01	43.32 ± 0.02
Final body weight (FBW, g)	83.70 ± 9.62 ^a^	102.35 ± 5.59 ^b^	102.53 ± 2.26 ^b^	104.15 ± 0.49 ^b^
Weight gain (WG, %)	92.50 ± 22.15 ^a^	136.1 ± 13.2 ^b^	137.6 ± 5.3 ^b^	140.5 ± 1.4 ^b^
Specific growth rate (SGR, %/day)	1.09 ± 0.19 ^a^	1.43 ± 0.10 ^b^	1.44 ± 0.04 ^b^	1.47 ± 0.01 ^b^
Feed conversion ratio (FCR)	1.03 ± 0.19	1.02 ± 0.08	0.92 ± 0.04	0.93 ± 0.04
Survival rate (SR, %)	100	100	100	100

^1^ Values (means ± SEM, n = 3) in the same row with different letter superscripts were significantly different (*p* < 0.05).

**Table 4:** Effects of three organic acids on the nutritional composition of largemouth bass (wet weight).

Items	CON	CA	FA	MA
Moisture/%	70.89 + 0.92	71.45 + 0.69	69.12 + 0.74	69.03 + 0.93
Crude protein/%	16.71 ± 0.27	17.37 ± 1.16	17.59 ± 0.96	17.92 ± 0.18
Crude lipid/%	7.72 ± 0.44	7.93 ± 0.69	8.22 ± 0.32	7.84 ± 0.89
Ash/%	6.02 ± 0.25 ^a^	6.83 ± 0.36 ^b^	7.10 ± 0.13 ^b^	7.26 ± 0.13 ^b^
Liver lipid/%	5.03 ± 0.41 ^b^	2.83 ± 0.21 ^a^	3.11 ± 0.36 ^a^	2.80 ± 0.55 ^a^
Hepatic glycogen/(mg/g)	113.69 ± 3.90 ^b^	75.35 ± 5.67 ^a^	64.55 ± 5.62 ^a^	82.26 ± 6.88 ^a^

Values (means ± SEM, n = 3) in the same row with different letter superscripts were significantly different (*p* < 0.05).

**Table 5:** Effects of three organic acids on serum biochemical indices of largemouth bass.

Items	CON	CA	FA	MA
TP (g/L)	29.78 ± 1.41 ^a^	34.94 ± 1.09 ^b^	35.07 ± 1.89 ^b^	36.57 ± 0.44 ^b^
GLU (mmol/L)	3.77 ± 1.23 ^b^	2.02 ± 0.30 ^a^	2.50 ± 0.68 ^b^	2.69 ± 0.35 ^b^
TG (mmol/L)	22.81 ± 0.58 ^c^	14.55 ± 0.32 ^a^	17.87 ± 1.56 ^b^	18.61 ± 0.71 ^b^
TC (mmol/L)	9.29 ± 0.07	10.66 ± 1.83	8.33 ± 0.82	10.28 ± 0.46
AST (U/L)	29.83 ± 1.45 ^c^	17.81 ± 1.04 ^a^	22.35 ± 1.21 ^b^	20.25 ± 1.18 ^ab^
ALT (U/L)	9.59 ± 0.27 ^c^	6.40 ± 0.16 ^a^	7.58 ± 0.18 ^b^	6.89 ± 0.12 ^a^
AKP (U/L)	131.5 ± 14.31 ^a^	175.81 ± 10.51 ^b^	198.15 ± 12.01 ^b^	220.47 ± 9.43 ^b^
SOD (U/mL)	268.54 ± 15.12 ^a^	336.24 ± 17.29 ^b^	308.99 ± 14.42 ^b^	311.71 ± 14.46 ^b^
CAT (U/mL)	6.84 ± 0.29 ^a^	8.57 ± 0.54 ^b^	8.04 ± 0.94 ^b^	8.13 ± 0.80 ^b^
MDA (nmol/mL)	10.72 ± 0.61 ^b^	8.38 ± 0.93 ^a^	8.79 ± 0.83 ^a^	8.64 ± 0.38 ^a^

TP: total protein; GLU: glucose; TG: triglyceride; TC: total cholesterol; ALT: alanine aminotransferase; AST: aspartate aminotransferase; AKP: alkaline phosphatase; SOD: superoxide dismutase; CAT: catalase; MDA: malondialdehyde. Values (means ± SEM, n = 3) in the same row with different letter superscripts were significantly different (*p* < 0.05).

## Data Availability

The original contributions presented in this study are included in the article/Supplementary Material. Further inquiries can be directed to the corresponding author(s).

## Supplementary Materials

The following supporting information can be downloaded at: https://www.mdpi.com/article/10.3390/ani16081198/s1. Table S1: Completed raw data summary for the organic acids supplementation experiment in largemouth bass.
